# Intravenous dosing of tocilizumab in patients younger than two years of age with systemic juvenile idiopathic arthritis: results from an open-label phase 1 clinical trial

**DOI:** 10.1186/s12969-019-0364-z

**Published:** 2019-08-22

**Authors:** Navita L. Mallalieu, Sunethra Wimalasundera, Joy C. Hsu, Wendy Douglass, Chris Wells, Inmaculada Calvo Penades, Ruben Cuttica, Hans-Iko Huppertz, Rik Joos, Yukiko Kimura, Diana Milojevic, Margalit Rosenkranz, Kenneth Schikler, Tamas Constantin, Carine Wouters

**Affiliations:** 1 Roche Innovation Center, 430 East 29th Street, New York, NY 10016 USA; 2 Roche Products Ltd., Welwyn Garden City, UK; 3 Unidad de Reumatología Pediátrica, Hospital Universitario y Politécnico La Fe, Valencia, Spain; 4 Sección Reumatologia, Hospital General de Niños Pedro de Elizalde, Buenos Aires, Argentina; 5 Center for Paediatrics and Youth Medicine, Professor Hess Children’s Hospital, Bremen, Germany; 6 Department of Rheumatology, ZNA/UZ, Antwerp/Gent, Belgium; 7 Division of Pediatric Rheumatology, Joseph M. Sanzari Children’s Hospital, Hackensack University Medical Center, Hackensack, NJ USA; 8 Pediatric Rheumatology, Johns Hopkins All Children’s Department of Medicine, St. Petersburg, FL USA; 9 Division of Pediatric Rheumatology, UPMC Children’s Hospital of Pittsburgh, Pittsburgh, PA USA; 10 Department of Pediatrics, University of Louisville Medical School, Louisville, KY USA; 11 Department of Pediatrics, Semmelweis University, Budapest, Hungary; 12 Department of Microbiology and Immunology, Laboratory Immunobiology and University Hospitals Leuven, Pediatric Rheumatology, Katholieke Universiteit Leuven, University of Leuven, Leuven, Belgium

**Keywords:** Biological therapy, Inflammation, Juvenile idiopathic arthritis, Pharmacokinetics

## Abstract

**Background:**

The anti–interleukin-6 receptor-alpha antibody tocilizumab was approved for intravenous (IV) injection in the treatment of patients with systemic juvenile idiopathic arthritis (sJIA) aged 2 to 17 years based on results of a randomized controlled phase 3 trial. Tocilizumab treatment in systemic juvenile idiopathic arthritis (sJIA) patients younger than 2 was investigated in this open-label phase 1 trial and compared with data from the previous trial in patients aged 2 to 17 years.

**Methods:**

Patients younger than 2 received open-label tocilizumab 12 mg/kg IV every 2 weeks (Q2W) during a 12-week main evaluation period and an optional extension period. The primary end point was comparability of pharmacokinetics during the main evaluation period to that of the previous trial (in patients aged 2–17 years), and the secondary end point was safety; pharmacodynamics and efficacy end points were exploratory. Descriptive comparisons for pharmacokinetics, pharmacodynamics, safety, and efficacy were made with sJIA patients aged 2 to 17 years weighing < 30 kg (*n* = 38) who received tocilizumab 12 mg/kg IV Q2W in the previous trial (control group).

**Results:**

Eleven patients (mean age, 1.3 years) received tocilizumab during the main evaluation period. The primary end point was met: tocilizumab exposures for patients younger than 2 were within the range of the control group (mean [±SD] μg/mL concentration at the end-of-dosing interval [C_min_]: 39.8 [±14.3] vs 57.5 [±23.3]; maximum concentration [C_max_] postdose: 288 [±40.4] vs 245 [±57.2]). At week 12, pharmacodynamic measures were similar between patients younger than 2 and the control group; mean change from baseline in Juvenile Arthritis Disease Activity Score-71 was − 17.4 in patients younger than 2 and − 28.8 in the control group; rash was reported by 14.3 and 13.5% of patients, respectively. Safety was comparable except for the incidence of serious hypersensitivity reactions (27.3% in patients younger than 2 vs 2.6% in the control group).

**Conclusions:**

Tocilizumab 12 mg/kg IV Q2W provided pharmacokinetics, pharmacodynamics, and efficacy in sJIA patients younger than 2 comparable to those in patients aged 2 to 17 years. Safety was comparable except for a higher incidence of serious hypersensitivity events in patients younger than 2 years.

**Classification:**

Juvenile idiopathic arthritis.

**Trial registration:**

ClinicalTrials.gov, NCT01455701. Registered, October 20, 2011,

Date of enrollment of first participant: October 26, 2012.

**Electronic supplementary material:**

The online version of this article (10.1186/s12969-019-0364-z) contains supplementary material, which is available to authorized users.

## Background

Systemic juvenile idiopathic arthritis (sJIA) has historically been the most severe and difficult to treat form of childhood arthritis, and treatment options were limited in the past [1, 2]. Approximately 5 to 15% of children with chronic arthritis in North America and Europe have sJIA, which is distinct from other categories of juvenile idiopathic arthritis (JIA) and is characterized by chronic arthritis with systemic manifestations and increased inflammatory markers [2–5]. Greater understanding of the biology of sJIA has led to improvements in outcomes for patients with sJIA resulting from the development of cytokine-targeted therapies [4]. The inflammatory cytokine interleukin-6 (IL-6) plays a pathogenic role in sJIA [1, 6, 7]. Tocilizumab (TCZ) is a humanized anti-human IL-6 receptor-alpha (IL-6Rα) monoclonal antibody that blocks IL-6 signaling [8, 9]. TCZ is indicated for the treatment of sJIA and polyarticular JIA (in patients 2–17 years of age), rheumatoid arthritis, giant cell arteritis [9, 10], and chimeric antigen receptor T-cell–induced cytokine release syndrome [9].

In the phase 3 TENDER study, patients 2 to 17 years of age with active sJIA and inadequate responses to nonsteroidal anti-inflammatory drugs (NSAIDs) and glucocorticoids were randomly assigned to receive either TCZ or placebo intravenously (IV) every 2 weeks (Q2W) double-blind for 12 weeks [1]. Patients who weighed less than 30 kg received TCZ 12 mg/kg, whereas patients who weighed ≥30 kg received TCZ 8 mg/kg. Because body weight influences exposure to TCZ, the difference in dosing regimens was necessary to provide comparable TCZ exposures for patients across the full range of body weights examined. Results demonstrated that inhibition of IL-6 with TCZ was efficacious in patients with severe, persistent, and unresponsive sJIA and that adverse events (AEs) were consistent with the known safety profile of TCZ. Results of the TENDER study formed the basis for United States Food and Drug Administration (FDA) and European Medicines Agency (EMA) approval of IV TCZ in 2011 for the treatment of patients with sJIA 2 years of age or older.

The current study was conducted to investigate TCZ treatment in patients with sJIA younger than 2 years and is the first trial to investigate a biologic therapy in children of this age with sJIA. Pharmacokinetics, safety, pharmacodynamics, and efficacy of TCZ were investigated and compared with data from patients in the TENDER trial who weighed < 30 kg and received the same TCZ dosing regimen of 12 mg/kg IV Q2W.

## Patients and methods

### Trial design

This trial in patients with sJIA younger than 2 years (ClinicalTrials.gov, NCT01455701) was an open-label, single-arm study with a 12-week main evaluation period and an optional extension period that lasted from week 12 until the patient reached 2 years of age or received treatment for 1 year, whichever was longer. In the previous TENDER study in patients with sJIA aged 2 to 17, patients received TCZ IV Q2W at a dose of 8 mg/kg if they weighed ≥30 kg or 12 mg/kg if they weighed < 30 kg [1]. Because it was highly likely that patients younger than 2 years would weigh < 30 kg, a TCZ dose of 12 mg/kg IV Q2W was selected for the current study.

After a screening period of up to 3 weeks, eligible patients received a maximum of 6 doses of TCZ 12 mg/kg IV Q2W from baseline to week 10 during the main evaluation period. Final evaluations occurred at week 12. After completing the main evaluation period, patients could enter the optional extension period and continue to receive TCZ 12 mg/kg IV Q2W if it was medically warranted. Patients who did not enter the optional extension period underwent safety follow-up 4 and 8 weeks after their last TCZ infusion. Efficacy and safety results of the main evaluation period and safety results after completion of the optional extension period of this trial are reported. Results from this trial were compared with those of patients aged 2 to 17 years who weighed < 30 kg and received TCZ 12 mg/kg IV Q2W in the TENDER trial (control group).

### Patients

Eligible patients were younger than 2 years, received a diagnosis of sJIA based on the International League of Associations for Rheumatology classification criteria [3], had symptoms for ≥1 month before screening, and had uncontrolled sJIA despite treatment with glucocorticoids and NSAIDs. Patients had to have ≥2 active joints with or without fever (defined as ≥38 °C) attributed to sJIA. Patients could not have had macrophage activation syndrome (MAS) within 3 months before the screening visit or previous exposure to TCZ, and they had to discontinue any biologic treatment with an appropriate washout period. The study was conducted in accordance with the principles of the Declaration of Helsinki and Good Clinical Practice and was approved by the institutional review boards and/or ethics committees of the participating centers (Additional file 1: Appendix 1). Written informed consent to participate in the study was obtained from the patients’ parents or legal guardians.

### Assessments

The primary end point was characterization of pharmacokinetics during 12 weeks of TCZ treatment; comparison was made to week 12 results from patients in the control group of the TENDER study to investigate whether individual pharmacokinetic parameters in children younger than 2 years fell within the range reported for older children. The safety of TCZ in combination with stable, ongoing sJIA therapy through 12 weeks was a secondary end point. Pharmacodynamics and efficacy of TCZ for the 12-week treatment period were exploratory end points; pharmacodynamic measures were soluble IL-6R (sIL-6R) levels, C-reactive protein (CRP) levels, and erythrocyte sedimentation rate (ESR); Juvenile Arthritis Disease Activity Score-71 (JADAS-71) and systemic features (fever and rash from study diaries) were efficacy end points. Anti-TCZ antibody (ADA) and corresponding TCZ and sIL-6R concentrations at the same time points were measured in all patients to assess immunogenicity. Blood samples for ADA measurement were taken at baseline, week 12, end of study, time of withdrawal because of anaphylaxis or serious or nonserious hypersensitivity, and ≥ 6 weeks after the last TCZ dose for patients who withdrew because of anaphylaxis or hypersensitivity reactions (hypersensitivity reactions were classified as any AE reported within 24 h after initiation of the TCZ infusion with a relationship of not “unrelated” to TCZ).

### Statistical analysis

Descriptive statistics were produced for this study (see Additional file 1: Appendix 2 for sample size calculation). Analyses were conducted on all patients who had ≥1 serum pharmacokinetic sample with valid TCZ concentration data. A 2-compartment pharmacokinetic model with combined first-order and Michaelis-Menten elimination that was previously developed for TCZ in pediatric patients with data from the TENDER trial was used to derive individual post hoc pharmacokinetic parameters using the “POSTHOC” option in nonlinear mixed-effects modeling (NONMEM) software. Individual post hoc pharmacokinetic parameters were subsequently used to obtain individual exposure values (maximum observed postinfusion serum concentration [C_max_], concentration at the end of a dosing interval [C_min_], and systemic exposure—defined as area under the serum concentration–time profile during the 2-week dosing interval [AUC_2weeks_]) at steady state by simulation. Post hoc pharmacokinetic exposure parameters were used to compute individual patient C_min_, C_max_, and AUC_2weeks_ at week 12. Comparisons were performed for observed TCZ trough concentrations (C_trough_) and modeled data. GraphPad Prism was used to generate graphs for pharmacokinetic parameters. Efficacy (JADAS-71) analysis was performed using SAS 9.2 for tables and SAS 9.4 for graphs.

Safety was assessed for the entire study period in all enrolled patients who received ≥1 dose of study medication regardless of whether they withdrew from the study, and efficacy was assessed in all patients who received ≥1 dose of study medication and had ≥1 efficacy assessment.

## Results

### Patients

**Fig. 1:**
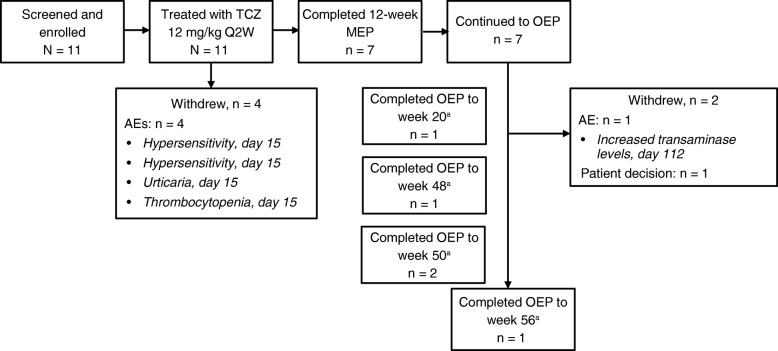
Patients younger than 2 years of age: patient disposition. ^a^Two patients completed the OEP upon reaching age 2, 1 at week 20 and 1 at week 56; 3 patients completed the OEP (last dose), 2 at week 50 and 1 at week 48. AE: adverse event; MEP: main evaluation period; OEP: optional extension period; Q2W: every 2 weeks; TCZ: tocilizumab

Eleven patients younger than 2 years were screened and enrolled from 11 centers (5 in the United States, 1 in Argentina, 5 in Europe); each center enrolled 1 patient. All patients received ≥1 dose of TCZ 12 mg/kg and were included in pharmacokinetic, safety, and efficacy analyses. Four patients withdrew from the main evaluation period because of AEs (3 because of clinically confirmed serious AEs of hypersensitivity and 1 because of an AE of thrombocytopenia). Seven patients completed the study to week 12 and entered the optional extension period, whereupon they all received ≥1 dose of 12 mg/kg TCZ IV Q2W (Fig. 1). Two patients withdrew prematurely from TCZ treatment during the optional extension period, 1 because of an AE and 1 because of withdrawal of consent. Baseline demographics and disease characteristics of patients younger than 2 years in this study and for the control group were as expected for the different age groups and disease durations (Table 1), though more of the patients younger than 2 years were female.

**Table 1:** Baseline demographics and disease characteristics

	Patients < 2 years of age < 30 kg (NP25737) *n* = 11	Patients 2–17 years of age (TENDER)^a^ < 30 kg *n* = 38
Age, years	1.3 (0.33)	6.6 (3.3)
Min, max	0.83, 1.83	2, 16
Females, n (%)	7 (63.6)	18 (47)
Weight, kg	9.97 (1.38)	20.07 (5.93)
Disease duration, years	0.319 (0.17)	4.03 (3.160)
JADAS-71, median (min, max)	20.50^b^ (9.0, 37.6)	32.70 (12.6, 96.1)
No. of active joints, median (min, max)	4.0 (2, 19)	13.5 (3, 71)
No. of joints with LOM, median (min, max)	3.0 (0, 15)	14.5 (0, 67)
Physician global VAS score, median (min, max)	62.0 (30, 98)	71.0 (28, 100)
ESR, mm/h	59.4^b^ (27.5)	64.1 (29.76)
CRP level, mg/L, median (min to max)	33.3 (18.0 to 1190.0)	123.2 (5.36 to 1704.9)
Fever, n (%)	5 (45.5)	20 (53.0)^c^
Rash, n (%)	8 (72.7)	13 (34.0)
No. of patients exposed to previous biologics, n (%)	2 (18.2)^d^	28 (73.7)
Previous MTX use, n (%)	2 (18.2)	31 (81.6)
Previous glucocorticoid use, n (%)	8 (72.7)	36 (94.7)

All values are mean (SD) unless otherwise noted

*CRP* C-reactive protein, *ESR* erythrocyte sedimentation rate, *IV* intravenous, *JADAS-71* Juvenile Arthritis Disease Activity Score in 71 joints, *LOM* limitation of movement, *MTX* methotrexate, *Q2W* every 2 weeks, *SD* standard deviation, *TCZ* tocilizumab, *VAS* visual analog scale

^a^Patients weighing < 30 kg, TCZ 12 mg/kg IV Q2W

^b^Efficacy-evaluable patients, *n* = 10

^c^Fever present for the past 7 days

^d^Two patients had received anakinra before study entry

Concomitant use

### Pharmacokinetics

**Fig. 2:**
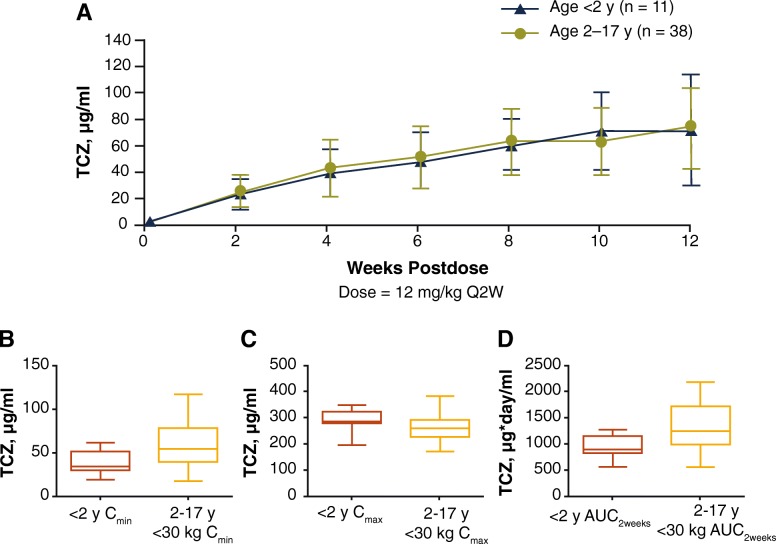
**a** Mean observed serum TCZ concentration–time profile in patients younger than 2 years of age and in patients 2 to 17 years of age and model-predicted steady state pharmacokinetic parameters in patients younger than 2 years and in patients 2 to 17 years of age for (**b**) C_min_, (**c**) C_max_, and (**d**) AUC_2weeks_. **a** Data are mean ± SD. **b-d** Data are medians and interquartile ranges (boxes) and minimum, maximum values (whiskers). Patients weighing < 30 kg and receiving TCZ 12 mg/kg IV Q2W were included from the study in patients 2 to 17 years of age (*n* = 38). AUC_2weeks_: area under the concentration–time curve to 2 weeks; C_max_: maximum observed postinfusion serum concentration; C_min_: concentration at the end of a dosing interval; IV: intravenous; Q2W: every 2 weeks; SD: standard deviation; TCZ: tocilizumab; y: year

There was a 3.3-fold increase in observed mean C_trough_ from week 2 through week 12 of the treatment period in patients younger than 2 years, which was consistent with that observed in the control group from the previous study (3.1-fold increase over 12 weeks in patients in the control group) (Fig. 2a). After TCZ administration, mean [±SD] observed TCZ C_trough_ increased over time and plateaued between weeks 10 (68.4 [±29.2] μg/mL) and 12 (69.2 [±42.0] μg/mL). The observed mean [SD] C_trough_ achieved at 12 weeks in patients younger than 2 years was comparable to that achieved in the control group (70.6 [±30.6] μg/mL; *n* = 30). Similarly, model-predicted steady state mean (median [range]) TCZ exposures observed in patients younger than 2 years were within the range of exposures in the older children: C_min_, 39.8 (34.3 [19.2 to 61.7]) μg/mL vs 57.5 (54.3 [10.9 to 117]) μg/mL; C_max_, 288 (285 [195 to 347]) μg/mL vs 245 (244 [109 to 382])] μg/mL; AUC_2weeks_, 947 (896 [563 to 1273]) μg/mL·day vs 1341 (1279 [558 to 2412]) μg/mL·day) (Fig. 2b).

### Pharmacodynamics

**Fig. 3:**
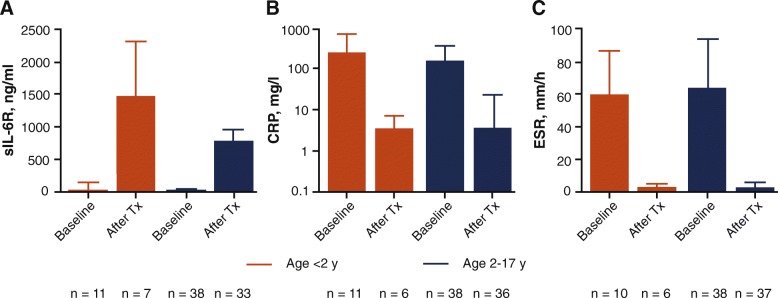
Mean observed serum concentrations at baseline and week 12 in patients younger than 2 years of age and in patients 2 to 17 years of age for **a** sIL-6R, **b** CRP, and **c** ESR. Data are mean ± SD. Patients younger than 2 years (*n* = 11); patients weighing < 30 kg and receiving TCZ 12 mg/kg IV Q2W were included from the study in patients 2 to 17 years of age (*n* = 38). ESR: upper limit of normal, 20 mm/h; CRP: upper limit of normal, generally considered to be 0–4.9 mg/L in adults. CRP: C-reactive protein; ESR: erythrocyte sedimentation rate; IV, intravenous; Q2W: every 2 weeks; sIL-6R: soluble interleukin-6 receptor; SD: standard deviation; TCZ: tocilizumab; Tx: treatment; y: year

After the administration of TCZ in patients younger than 2 years, mean [±SD] observed serum sIL-6R levels increased, consistent with increasing TCZ concentrations, and reached steady state at week 8 (Fig. 3a, Additional file 2: Fig. S1). Levels of sIL-6R in patients younger than 2 years (927 [±148] ng/mL) were generally comparable to those of patients in the control group (770 [204] ng/mL) (Additional file 2: Fig. S1). Outlier values for serum sIL-6R concentrations at week 12 were observed in 2 of 11 patients younger than 2 years (2590 ng/mL and 2720 ng/mL).

At baseline, mean CRP and ESR (Fig. 3b, c) values were 251 mg/L and 59.4 mm/h, respectively, and decreased rapidly after the first dose of TCZ. By week 12, mean CRP and ESR values were within the normal range (Fig. 3b, c). Overall, week 12 sIL-6R, CRP, and ESR values were comparable to those for the control group.

### Efficacy

JADAS-71 and component values improved during the main evaluation period in patients younger than 2 years (Additional file 3: Fig. S2). The median JADAS-71 was 20.5 at baseline, decreased to near minimal disease activity (< 3.80) by day 57, and was at minimal disease activity (< 3.80) by day 85 (week 12). The percentage of patients with fever or rash decreased over the course of the study from baseline to week 12 (Table 2). The decrease in fever and rash was rapid, with fever resolving in 70% (7/10) of patients and rash resolving in 50% (5/10) of patients as early as day 8, after the first dose. The reduction in disease activity (median [minimum, maximum]) in patients younger than 2 years from baseline to week 12 (20.5 [9.0, 37.6] to 2.90 [0.3, 16.1]) was comparable to that observed for the control group at week 12 (32.7 [12.6, 96.1] to 6.15 [0.5, 20.8]) (Table 2). Reductions in individual JADAS-71 components and number of joints with limitation of movement were also similar between patients younger than 2 years and the control group (Table 2). Oral corticosteroid dose was stable, then decreased by approximately 40% from week 8 to week 12, for patients younger than 2 years (Additional file 4: Fig. S3), consistent with the decrease in corticosteroid dose observed in the control group [1].

**Table 2:** Change from baseline to week 12 in JADAS-71

	Patients < 2 years of age < 30 kg *n* = 11	Patients 2–17 years of age^a^ < 30 kg *n* = 38
JADAS-71^b^		
Baseline	20.5 (9.0 to 37.6), *n* = 10	32.7 (12.6 to 96.1), *n* = 38
Change from baseline to week 12	−13.9 (− 2.7 to − 10.1), *n* = 5^c^	−25.8 (− 91.2 to 0.0), *n* = 38
No. of joints with LOM		
Baseline	3.0 (0 to 15), *n* = 11	14.5 (0 to 67), *n* = 38
Change from baseline to week 12	−3.0 (− 9 to 0), *n* = 7	− 8.0 (− 6 to 28), *n* = 37
JADAS-71 component^b^		
Physician global VAS score, mm		
Baseline	62.0 (30 to 98), *n* = 11	71.0 (28 to 100), *n* = 38
Change from baseline to week 12	−44.0 (− 69 to − 37), *n* = 7	− 50.0 (− 99 to − 9), *n* = 37
Parent global VAS score, mm		
Baseline	55.0 (10 to 99), *n* = 11	65.5 (8 to 100), *n* = 38
Change from baseline to week 12	−28.0 (− 85 to − 21), *n* = 7	− 42.0 (− 100 to − 6), *n* = 37
No. of active joints		
Baseline	4.0 (2 to 19), *n* = 11	13.5 (3 to 71), *n* = 38
Change from baseline to week 12	−7.0 (− 16 to − 2), *n* = 7	− 10.0 (− 68 to − 1), *n* = 37
Fever and rash		
Presence of fever, n/N (%)		
Baseline	9/11 (81.8)	26/38 (68.4)
Week 12	1/7 (14.3)	1/37 (2.7)
Presence of rash, n/N (%)		
Baseline	8/11 (72.7)	13/38 (34.2)
Week 12	1/7 (14.3)	5/37 (13.5)
Acute-phase reactants		
CRP level, mg/L		
Baseline	33.3 (18.0 to 1190.0), *n* = 11	123.2 (5.36 to 1704.9), *n* = 38
Change from baseline to week 12	− 74.1 (1181.0 to − 13.0), *n* = 6	− 123.1 (−1704.6 to −5.23), *n* = 36
ESR, mm/h		
Baseline	57.0 (10.0 to 100.0), *n* = 10	69.0 (8 to 130), *n* = 38
Change from baseline to week 12	− 59.0 (− 78.0 to − 6.0), *n* = 5	− 65.0 (− 126 to − 7), *n* = 37

All values are median (min to max) unless otherwise noted

*CRP* C-reactive protein, *ESR* erythrocyte sedimentation rate, *IV* intravenously, *JADAS-71* Juvenile Arthritis Disease Activity Score in 71 joints, *LOM* limitation of movement, *Q2W* every 2 weeks, *TCZ* tocilizumab, *VAS* visual analog scale

^a^Patients weighing < 30 kg and receiving 12 mg/kg TCZ IV Q2W includes patients who were receiving placebo at baseline and switched to TCZ after week 12

^b^Efficacy-evaluable patients

^c^Patients who did not withdraw

### Safety

#### Main evaluation period

During the main evaluation period of the study, most patients younger than 2 years had ≥1 AE (10/11 patients; 90.9%). The nature of AEs was comparable between the age groups in both studies (Table 3); however, a higher percentage of patients younger than 2 years experienced AEs that led to withdrawal (3 because of clinically confirmed serious AEs of hypersensitivity and 1 because of a nonserious AE of thrombocytopenia). During the main evaluation period, 3 of 11 (27.3%) patients experienced SAEs; 2 patients reported 1 SAE each (hypersensitivity and urticaria), both of which were considered by the investigator to be related to TCZ treatment and led to study discontinuation. One patient reported 3 SAEs (hypersensitivity, hand-foot-and-mouth disease, and JIA flare); only hypersensitivity was considered by the investigator to be related to TCZ treatment and led to withdrawal; the other 2 SAEs occurred during the safety follow-up period. There were no other serious infections during the main evaluation period.

**Table 3:** Safety

Adverse events	Patients < 2 years of age < 30 kg *n* = 11		Patients 2–17 years of age^a^ < 30 kg *n* = 38
	12-week MEP	Entire study	First 12 weeks
Patients with ≥1 AE			
AE	10 (90.9)	10 (90.9)	33 (86.8)
SAE	3 (27.3)	5 (45.5)	2 (5.3)
AE with fatal outcome	0	0	0
AE leading to withdrawal	4 (36.4)	5 (45.5)	1 (2.6)
AE leading to dose interruption	1 (9.1)	5 (45.5)	4 (10.5)
Infection			
AE	9 (81.8)	9 (81.8)	23 (60.5)
SAE	1 (9.1)	1 (9.1)	1 (2.6)
Hypersensitivity reactions^b^			
Clinically confirmed	4 (36.4)	4 (36.4)	1 (2.6)
Serious	3 (27.3)	3 (27.3)	1 (2.6)
Low neutrophil count (grade ≥ 3)	1 (9.1)	3 (27.3)	3 (7.9)
Low platelet count (grade ≥ 3)	1 (9.1)	0	0

All data are number (%) of patients with event

*AE* adverse event, *IV* intravenously, *MEP* main evaluation period, *Q2W* every 2 weeks, *SAE* serious adverse event, *TCZ* tocilizumab

^a^Patients weighing < 30 kg and receiving TCZ 12 mg/kg IV Q2W

^b^See Additional file 1: Appendix 3 for full details of patients with hypersensitivity reactions

There were 4 clinically confirmed hypersensitivity events in the main evaluation period (Table 3). One patient experienced mild, nonserious urticaria after the day 1 TCZ infusion, and 3 patients experienced serious hypersensitivity reactions during or immediately after the day 15 TCZ infusion (2 hypersensitivity, 1 urticaria) that led to withdrawal. The 2 serious events of hypersensitivity involved multiple signs and symptoms and were associated with confounding factors: in 1 patient, an administration error of faster infusion rate occurred; in the other, a concomitant diagnosis of subclinical MAS was made (Additional file 1: Appendix 3). All 4 confirmed hypersensitivity events resolved without sequelae after treatment.

Three patients who tested negative for anti-TCZ antibodies at baseline tested positive for anti-TCZ antibodies after TCZ treatment during the main evaluation period. These patients were at the lower end of the predose TCZ exposure range at day 15 (Additional file 5: Fig. S4) and were withdrawn from the study because of AEs (2 hypersensitivity, 1 thrombocytopenia) on day 15 after their second TCZ infusion. These patients received only 2 doses; therefore, efficacy could not be adequately assessed.

#### Total observation period (main evaluation period and optional extension period)

Throughout the course of the study (main evaluation period and optional extension period) in patients younger than 2 years, most (90.9%; 10/11) were reported to have ≥1 AE (Table 3). SAEs were reported by 5 of 11 patients (45.5%). Two occurred during the optional extension period: 1 patient had increased transaminase levels, considered by the investigator to be related to treatment with both TCZ and concomitant methotrexate, and was withdrawn from the optional extension period; another patient experienced MAS (reported as hemophagocytic histiocytosis), which was considered unrelated to TCZ treatment and resolved after temporary dose interruption and treatment with methylprednisone. Neither of these patients had other clinical symptoms of MAS. No serious infections were reported during the optional extension period; hence, the total number of serious infections during the total observation period was the single event of hand-foot-and-mouth disease reported in the safety follow-up after withdrawal from the main evaluation period. No clinically confirmed hypersensitivity reactions were reported in the optional extension period; therefore, during the total observation period, 4 (36.4%) clinically confirmed events were reported. During the entire study, AEs leading to dose interruption occurred in 5 of 11 patients (1 in the main evaluation period, 4 in the optional extension period) (Table 3), primarily because of infections, neutropenia, and elevated liver enzymes, all mild or moderate in intensity. No deaths in patients younger than 2 years occurred during this study.

## Discussion

Until recent years, treatment of sJIA was challenged by limited therapeutic options. However, the outcome for patients with sJIA has substantially improved since the development of cytokine antagonists. The phase 3 TENDER study of TCZ in patients with sJIA resulted in FDA and EMA approval of IV TCZ for the treatment of patients 2 years or older, but sJIA can be diagnosed in even younger patients. Very young patients have been shown to exhibit more severe inflammatory features and a risk for worse outcomes [11]. To allow rational interpretation of data from this study of a small population of patients with a severe disease, the focus of the research was to facilitate a comparison of key measures influencing the well-characterized efficacy of TCZ. It was anticipated that comparable results in pharmacokinetics would likely translate to comparable outcomes in pharmacodynamics, safety, and efficacy. Therefore, this open-label study investigated the pharmacokinetics, safety, pharmacodynamics, and efficacy of IV TCZ in children with sJIA younger than 2 years. The most relevant control group for comparison was the subgroup of patients from the TENDER study whose body weight was below the same cutoff (< 30 kg) and who received the same dosing regimen (12 mg/kg TCZ IV Q2W). Although body size has been identified as the key demographic factor influencing exposure to TCZ [12], it is possible that ontogenic factors (such as age and maturation-related factors) influence exposure to TCZ, as they do with other monoclonal antibodies [13]. Data from infants treated with monoclonal antibodies are limited; results from the current trial provide the first pharmacokinetic exposure data for a biologic in patients with sJIA in this age range. During the 12-week main evaluation period of the study in patients with sJIA younger than 2 years, the 12 mg/kg TCZ IV Q2W regimen resulted in pharmacokinetic, pharmacodynamic, and efficacy results that were consistent with week 12 results for the control group of older patients with sJIA treated with the same regimen. Although no direct evidence rules out differences in the etiology of the disease between the age groups, the data generated in this study indicate consistency in exposure and efficacy between patients younger than 2 years and the population of patients 2 years and older. All patients in the current study achieved reductions in disease activity from week 12 to baseline—measured by number of patients with fever and systemic inflammation, number of joints with limitation of movement, and JADAS-71 scores—comparable to those for older children in the control group. Results observed for pharmacodynamic parameters (sIL-6R, CRP, ESR) were comparable between patients younger than 2 years and the control group of older patients. Pharmacokinetic exposures obtained in this study in patients younger than 2 years led to the expected pharmacodynamic response in that sIL-6R builds to a plateau after repeated dosing with TCZ as it forms a complex with TCZ [14].

The safety profile for 12 mg/kg TCZ IV Q2W during 12 weeks of treatment in this study was similar between patients younger than 2 years and the control group of patients aged 2 to 17 years, except for a higher incidence of serious hypersensitivity events in patients younger than 2 years. All 3 serious hypersensitivity events occurred during or immediately after the second TCZ infusion (day 15). Two of the patients who had serious hypersensitivity events developed ADAs, detected after day 15. Another patient who was positive for ADA after day 15 did not experience any clinically confirmed hypersensitivity events. Therefore, in this small sample of 11 patients, there does not appear to be a relationship between ADA and hypersensitivity reactions, but such a relationship cannot be definitively ruled out because of the small sample size.

Safety results from the optional extension period of this study in sJIA patients younger than 2 years were generally consistent with those of the main evaluation period, and no additional safety signals were identified other than the higher incidence of serious hypersensitivity in the main evaluation period. During the course of the study, most AEs were mild or moderate in intensity, and the types of AEs observed were consistent with the known safety profile of TCZ for sJIA. During the optional extension period, TCZ treatment was well tolerated in patients younger than 2 years, and no additional safety signals were noted. However, because of the small sample size and the confounding clinical factors for 2 of the 3 serious hypersensitivity events that occurred in the main evaluation period (faster than normal TCZ infusion rate and subclinical MAS [15]), no definitive conclusions can be made regarding the safety of TCZ IV for patients with sJIA younger than 2 years. Other limitations of the current study of TCZ in patients with sJIA younger than 2 years include the lack of a placebo comparator and the lack of statistical comparisons.

## Conclusion

In conclusion, the dosing regimen of 12 mg/kg IV TCZ Q2W provided pharmacokinetics, pharmacodynamics, and efficacy data in sJIA patients younger than 2 years comparable to those in patients 2 years of age or older. Safety was comparable except for a higher incidence of serious hypersensitivity events in the younger patients.

### Previous presentation

The data in this manuscript were presented at the following congresses:

- EULAR 2018 (European League Against Rheumatism); June 13–16, 2018; Amsterdam, Netherlands – Mallalieu NL et al. *Ann Rheum Dis* 2018;77(suppl). Abstract 102.
- 2018 ACR/ARHP (American College of Rheumatology); October 19–24, 2018; Chicago, Illinois – Wimalasundera S et al. *Arthritis Rheumatol* 2018;70(suppl 10). Abstract 1413.

## Additional files

Additional file 1: **Appendix 1.** List of investigators and institutional review boards or ethics committees. **Appendix 2.** Calculation of sample size. **Appendix 3.** Details of patients with hypersensitivity reactions. (DOCX 59 kb)

Additional file 2: **Fig. S1.** Mean observed serum sIL-6R concentration–time profile in patients younger than 2 years and in patients 2–17 years of age. (EPS 1812 kb)

Additional file 3: **Fig. S2.** JADAS-71 in patients younger than 2 years (< 30 kg). (EPS 1824 kb)

Additional file 4: **Fig. S3.** Mean corticosteroid dose by visit for sJIA in patients younger than 2 years (< 30 kg). (EPS 1849 kb)

Additional file 5: **Fig. S4.** Patients younger than 2 years (< 30 kg). Overlay of individual observed serum TCZ concentration profiles for patients with or without treatment-induced anti-TCZ antibodies. (EPS 1923 kb)

## Data Availability

Qualified researchers may request access to data through the clinical study data request platform (www.clinicalstudydatarequest.com). Further details on Roche’s criteria for eligible studies are available here (https://clinicalstudydatarequest.com/Study-Sponsors/Study-Sponsors-Roche.aspx). For further details on Roche’s Global Policy on the Sharing of Clinical Information and how to request access to related clinical study documents, see here (https://www.roche.com/research_and_development/who_we_are_how_we_work/clinical_trials/our_commitment_to_data_sharing.htm).

## Abbreviations

ADA: anti–tocilizumab antibody
AE: adverse event
AUC_2weeks_: area under the concentration–time curve to 2 weeks
C_max_: maximum observed postinfusion serum concentration
C_min_: model estimated tocilizumab concentration at the end of a dosing interval
CRP: C-reactive protein
C_trough_: observed tocilizumab trough concentration
EMA: European Medicines Agency
ESR: erythrocyte sedimentation rate
FDA: US Food and Drug Administration
IL-6Rα: interleukin-6 receptor alpha
IV: intravenous
JADAS-71: Juvenile Arthritis Disease Activity Score in 71 joints
JIA: juvenile idiopathic arthritis
LOM: limitation of movement
MAS: macrophage activation syndrome
MTX: methotrexate
NSAID: nonsteroidal anti-inflammatory drug
Q2W: every 2 weeks
SAE: serious adverse event
SD: standard deviation
sIL-6Rα: soluble interleukin-6 receptor alpha
sJIA: systemic juvenile idiopathic arthritis
TCZ: tocilizumab
Tx: treatment
VAS: visual analog scale

## References

[1] De Benedetti F, Brunner HI, Ruperto N, Kenwright A, Wright S, Calvo I (2012). Randomized trial of tocilizumab in systemic juvenile idiopathic arthritis. N Engl J Med.

[2] Giancane G, Minoia F, Davi S, Bracciolini G, Consolaro A, Ravelli A (2016). IL-1 inhibition in systemic juvenile idiopathic arthritis. Front Pharmacol.

[3] Petty RE, Southwood TR, Manners P, Baum J, Glass DN, Goldenberg J (2004). International league of associations for rheumatology classification of juvenile idiopathic arthritis: second revision, Edmonton, 2001. J Rheumatol.

[4] Grevich S, Shenoi S (2017). Update on the management of systemic juvenile idiopathic arthritis and role of IL-1 and IL-6 inhibition. Adolesc Health Med Ther.

[5] Yokota S, Tanaka T, Kishimoto T (2012). Efficacy, safety and tolerability of tocilizumab in patients with systemic juvenile idiopathic arthritis. Ther Adv Musculoskelet Dis.

[6] Yokota S, Kishimoto T (2010). Tocilizumab: molecular intervention therapy in children with systemic juvenile idiopathic arthritis. Expert Rev Clin Immunol.

[7] de Benedetti F, Massa M, Robbioni P, Ravelli A, Burgio GR, Martini A (1991). Correlation of serum interleukin-6 levels with joint involvement and thrombocytosis in systemic juvenile rheumatoid arthritis. Arthritis Rheum.

[8] Murakami M, Tomiita M, Nishimoto N (2012). Tocilizumab in the treatment of systemic juvenile idiopathic arthritis. Open Access Rheumatol.

[9] Actemra® (tocilizumab) injection, for intravenous or subcutaneous use. South San Francisco: Genentech, Inc.; 2019. https://dailymed.nlm.nih.gov/dailymed/drugInfo.cfm?setid=2e5365ff-cb2a-4b16-b2c7-e35c6bf2de13.

[10] RoActemra 162 mg solution for injection in pre-filled syringe. Welwyn Garden City. In: UK: Roche products limited; 2019. https://www.medicines.org.uk/emc/product/9086/smpc.

[11] Russo RA, Katsicas MM (2013). Patients with very early-onset systemic juvenile idiopathic arthritis exhibit more inflammatory features and a worse outcome. J Rheumatol.

[12] Frey N, Grange S, Woodworth T (2010). Population pharmacokinetic analysis of tocilizumab in patients with rheumatoid arthritis. J Clin Pharmacol.

[13] Malik P, Edginton A (2018). Pediatric physiology in relation to the pharmacokinetics of monoclonal antibodies. Expert Opin Drug Metab Toxicol.

[14] Uchiyama Y, Yoshida H, Koike N, Hayakawa N, Sugita A, Nishimura T (2008). Anti-IL-6 receptor antibody increases blood IL-6 level via the blockade of IL-6 clearance, but not via the induction of IL-6 production. Int Immunopharmacol.

[15] Ravelli A, Minoia F, Davi S, Horne A, Bovis F, Pistorio A (2016). 2016 classification criteria for macrophage activation syndrome complicating systemic juvenile idiopathic arthritis: a European league against rheumatism/American College of Rheumatology/Paediatric rheumatology international trials organisation collaborative initiative. Arthritis Rheum.

