# Supplementary material for: Intravenous dosing of tocilizumab in patients younger than two years of age with systemic juvenile idiopathic arthritis: results from an open-label phase 1 clinical trial
Source: Pediatr Rheumatol Online J. 2019 Aug 22;17:57. doi: 10.1186/s12969-019-0364-z (PMC6704523; doi:10.1186/s12969-019-0364-z)
Supplement: Supplementary file 1 — Appendix 1. List of investigators and institutional review boards or ethics committees. Appendix 2. Calculation of sample size. Appendix 3. Details of patients with hypersensitivity reactions. (DOCX 59 kb) [file 12969_2019_364_MOESM1_ESM.docx]

**Additional files**

**Additional file 1: Appendix 1.** List of investigators and institutional review boards or ethics committees.

| **Investigator** | **Institutional review board/ethics committee** | **Date of approval** |
| --- | --- | --- |
| Yukiko Kimura | Western International review board, Olympia, United States | January 11, 2012 |
| Kenneth Schikler | University of Louisville Human Subject Protection, Louisville, United States | January 19, 2012 |
| Margalit Rosenkranz | Western International review board, Olympia, United States | April 9, 2012 |
| Rubén Cuttica | Comité de Etica en Investgacion, Caba, Argentina | May 15, 2012 |
| Jonathan Bernstein | Western International review board, Puyallup, United States | August 8, 2012 |
| Diana Milojevic | Institutional review board Tufts Medical Center, Boston, United States | July 1, 2014 |
| Hans-Iko Huppertz | Ethikkommission des Landes Bremen, Bremen, Germany | August 10, 2015 |
| Carine Wouters | Commissie Medische Ethiek UZ, Leuven, Belgium | August 12, 2015 |
| Rik Joos | UZ Ghent Commissie voor Medische Ethiek, Ghent, Belgium | August 12, 2015 |
| Istvan Takacs | Medical Research Council Ethics Committee for Clinical Pharmacology, Budapest, Hungary | July 27, 2015 |
| Inmaculada Calvo Penades | Comité Etico de Investgacion Clinica, Valencia, Spain | June 15, 20105 |
| Inmaculada Calvo Penades | Hospita Ramon y Cajal Comité Etico de de Investigacion Clinica, Madrid, Spain | June 15, 2015 |

**Additional file 1: Appendix 2.** Calculation of sample size.

A sample size of ≥6 patients was calculated to be adequate for pharmacokinetic assessment based on the comparable percentage of coefficient of variation (CV%) in the various age groups with various sample sizes in the TENDER study. A sample size of 10 patients younger than 2 years of age was considered appropriate to confirm pharmacokinetic comparability with the TENDER study and to provide safety data for this age group. This sample size represents 13% of the sample size of 112 patients between 2 and 17 years of age in TENDER, 75 of whom were randomly assigned to receive tocilizumab. Furthermore, the number of patients in each 2-year age group category in TENDER ranged from 6 to 12 years, which is consistent with the sample size of 10 for patients younger than 2 years in the current study. Intersubject variability (coefficient of variation [CV%]) calculated in TENDER ranged from 23.1% to 35.7% for AUC_tau_, 30.6% to 47.2% for C_min_, and 16.6 to 33.7% for C_max_, demonstrating that inter-CV% values were consistent across age groups despite different sample sizes. It was estimated that 6 patients would provide adequate pharmacokinetic data in the study in patients younger than 2 years. The sample size of 10 was selected to confirm pharmacokinetic compatibility with data from TENDER and to provide safety data in patients younger than 2 years.

**Additional file 1: Appendix 3.** Details of patients with hypersensitivity reactions.

The 4 clinically confirmed hypersensitivity reactions in patients younger than 2 years occurred during or immediately after the TCZ infusion on day 1 (1 patient with nonserious urticaria was treated with intravenous diphenhydramine; urticaria resolved without sequelae on the same day, and the patient completed participation in the study) or on day 15 (1 patient with serious urticaria, which resolved after 2 days, but no other signs or symptoms of hypersensitivity; 1 patient [who had subclinical MAS] with serious and severe hypersensitivity symptoms of chills, rash, vomiting, fever, arterial hypotension, and tachycardia; and 1 patient mistakenly administered a faster TCZ infusion who experienced serious and severe hypersensitivity symptoms of chills, mottling skin, cold extremities, and decreased level of consciousness, all of which resolved without sequelae on the same day). All 3 patients with serious hypersensitivity reactions withdrew from the study in accordance with the protocol. In contrast, in the control group, 1 patient older than 2 had clinically confirmed serious hypersensitivity reactions (using the same criteria applied in the study in patients younger than 2 years): urticaria within 24 hours of the day 29 (week 4) infusion and angioedema during the day 57 (week 8) infusion.
